# 液相色谱-四极杆/飞行时间质谱法分析29种芬太尼类物质及其碎裂机理

**DOI:** 10.3724/SP.J.1123.2021.01036

**Published:** 2022-01-08

**Authors:** Zhenlin DONG, Chunguang YANG, Tian XU, Di DAI, Lu GAO, Lu YANG, Qiuyan WANG

**Affiliations:** 大连海关技术中心, 辽宁 大连 116001; Technical Center of Dalian Customs, Dalian 116001, China; 大连海关技术中心, 辽宁 大连 116001; Technical Center of Dalian Customs, Dalian 116001, China; 大连海关技术中心, 辽宁 大连 116001; Technical Center of Dalian Customs, Dalian 116001, China; 大连海关技术中心, 辽宁 大连 116001; Technical Center of Dalian Customs, Dalian 116001, China; 大连海关技术中心, 辽宁 大连 116001; Technical Center of Dalian Customs, Dalian 116001, China; 大连海关技术中心, 辽宁 大连 116001; Technical Center of Dalian Customs, Dalian 116001, China; 大连海关技术中心, 辽宁 大连 116001; Technical Center of Dalian Customs, Dalian 116001, China

**Keywords:** 液相色谱-四极杆/飞行时间质谱, 鉴别和确认, 碎裂机理, 筛查, 芬太尼类物质, liquid chromatography-quadrupole time-of-flight mass spectrometry (LC-QTOF-MS), identification and confirmation, fragmentation mechanism, screening, fentanyl analogs

## Abstract

芬太尼类物质品种繁多,自我国整类列管后,整类检测是该领域的重点和难点。该文详细研究了29种化合物的二级质谱碎片离子碎裂机理,总结出芬太尼类物质的碎裂规律和特点,为芬太尼物质的整类筛查检测提供参考。建立了分析29种芬太尼类物质的一级和二级质谱库的定性方法,建立了液相色谱-四极杆/飞行时间质谱(LC-QTOF-MS)检测29种芬太尼类物质的定量方法。药品和白色粉末类、蛋白质和乳饮料类样品经乙腈提取,含糖固体或粉末类、饮用水类、果蔬饮料类、保健饮料类、茶饮料类、酒类样品经10%乙腈水溶液提取,提取液经涡旋、离心和过膜后,采用Kinetex C18色谱柱(100 mm×2.1 mm, 2.6 μm)分离,以乙腈和0.08%甲酸水溶液为流动相进行梯度洗脱,采用四极杆/飞行时间质谱,在正离子模式下,外标法定量检测。结果表明,29种芬太尼类物质在1~20 μg/L范围内线性关系良好,相关系数均大于0.995,检出限(LOQ)均为0.01 mg/kg,定量限(LOQ)均为0.05 mg/kg,在降糖药、露露、葡萄糖粉、珍露保健饮料和巧克力样品中3个加标水平平均回收率为85.2%~112.9%,相对标准偏差(RSD)为1.9%~19.8%(*n*=6)。该方法操作简单,耗时短,灵敏度高,稳定性好,检测品种覆盖范围广,适用于药品类、含糖固体或粉末类、饮料类、饮用水类和酒类等样品中29芬太尼类物质的定性和定量检测。

## Abstract (en)

Given the wide variety of fentanyl analogs, the test for this entire group tends to be crucial and particularly difficult since all fentanyl-like substances are listed as controlled substances in China. This study meticulously analyzed the fragmentation pathways and mechanisms of 29 fentanyl analogs and summarized the fragmentation pathways and features for the entire group of fentanyl analogs, thus providing a reference for related screening tests. Fentanyl, thiofentanyl, and sufentanil were selected as the representative compounds in this study, and the fragmentation mechanism of their fragment ions was interpreted. The general fragmentation rules for fentanyl analogs were summarized as well. The fragment ions of the three compounds formed by induced cleavage (i) came with high abundance ratios, such as fragment ions of *m/z* 188, 105, 194, 111, and 238, while the induced cleavage was due to the amide and piperidinyl groups. Moreover, the induction ability of amide group was significantly stronger than that of the piperidinyl group, and induced cleavage was the main fragmentation pathway for most of the fentanyl analogs. Furthermore, the fragment ions with *m/z* 281 and 287 for fentanyl and thiofentanyl were formed by loss of the propionyl group after single H rearrangement (rH). The fragment ions with *m/z* 216, 146, and 132 for fentanyl and thiofentanyl were formed by double H rearrangement (r2H). Although their abundance ratios were not high, they still had specificity and regularity. Elimination reaction (re) was also a very common fragmentation pathway for these compounds, leading to fragment ions with *m/z* 134 and 140. Phenylethyl substituents were more prone to the elimination reaction with a higher abundance ratio than thiophenethyl substituents. Compounds such as sufentanil with methoxy substituents at the piperidinyl *para*-position could produce a large number of fragment ions, which were more susceptible to the rH pathway and loss of methanol neutral molecules, leading to the formation of ions with *m/z* 355. Similarly, compounds such as remifentanil bearing a methyl formate substituent at the piperidine *para*-position also produced numerous fragment ions, which were more prone to the rH pathway to lose methyl formate or methanol neutral molecules and furnish fragment ions with *m/z* 317 or 345. Compounds containing hydroxyl substituents, such as *β*-hydroxyfentanyl and *β*-hydroxythiofentanyl, produce significant dehydration ions and formed fragment ions with *m/z* 335 (*β*-hydroxyfentanyl) and *m/z* 341 (*β*-hydroxythiofentanyl). A method based on liquid chromatography-quadrupole time-of-flight mass spectrometry (LC-QTOF-MS) for the qualitative and quantitative determination of the 29 fentanyl analogs was developed. Drugs and white powder samples were extracted by acetonitrile, as well as protein and milk beverage samples. Sugar-containing solids or powders, drinking water, fruit and vegetable drinks, health drinks, tea drinks, and alcohol samples were extracted by 10% acetonitrile aqueous solution. Following vortexing, centrifugation, and membrane separation, the target compounds were separated on a Kinetex C18 column (100 mm×2.1 mm, 2.6 μm) with gradient elution at a flow rate of 0.4 mL/min. The mobile phases were composed of acetonitrile and 0.08% formic acid aqueous solution. The target compounds were quantified by LC-QTOF-MS using an external standard method in positive ion mode. The 29 fentanyl analogs showed good linear relationships in the range of 1-20 μg/L, and the correlation coefficients were greater than 0.995. The limits of detection (LODs) and limits of quantification (LOQs) were 0.01 mg/kg and 0.05, respectively. The average recoveries were 85.2%-112.9% for hypoglycemic drugs, Lulu drinks, glucose powder, Zhenlu health drink and chocolate, with RSDs of 1.9%-19.8% (*n*=6). This method is rapid, simple, time-saving, highly sensitivity and stable, and it is applicable to a wide variety of samples. Hence, it is suitable for the identification, confirmation, and quantitative detection of the 29 fentanyl analogs in drugs, solids or powders containing sugar, beverages, drinking water, wine samples, etc.

芬太尼(fentanyl)是一种人工合成的强效麻醉性阿片类镇痛药,属于新精神活性物质,其衍生物种类繁多,在2019年5月前,我国对25种芬太尼类物质和2种前体物质*N*-苯乙基-4-哌啶酮(NPP)和4-苯胺基-*N*-苯乙基哌啶(4-ANPP)实行管控。随着芬太尼衍生品种的不断增加,我国自2019年5月1日起将芬太尼类物质整类列入《非药用类麻醉药品和精神药品管制品种增补目录》中进行管控。

目前,芬太尼类物质的检测方法有酶联免疫吸附测定法(ELISA)、高效液相色谱法(HPLC)^[1,2]^、气相色谱-质谱法(GC-MS)^[3,4]^、液相色谱-串联质谱法(LC-MS/MS)^[5,6,7,8,9,10,11,12,13,14,15,16,17,18,19,20]^及液相色谱-高分辨质谱法(LC-HRMS)^[21,22,23,24,25,26,27]^等。HPLC灵敏度低,定性能力不足。GC-MS受检测方法限制,前处理繁琐,定性手段单一。LC-MS/MS应用较普遍,报道也较多,缺点是定性手段单一,疑似阳性样品尚需其他手段确证,罗耀等^[15]^采用LC-MS/MS检测固体和液体样品中27种芬太尼,Strayer等^[19]^采用LC-MS/MS检测血液中21种芬太尼类似物和代谢物,检测品种虽然较多,但存在仍需其他手段辅助确证的缺点。HPLC-HRMS检测芬太尼类物质既有稳定的定量能力,同时又兼具良好的鉴别和确证能力。张伟亚等^[23]^采用HPLC-HRMS无标准品快速筛查32种芬太尼,邓慧芬等^[27]^采用HPLC-HRMS检测27种芬太尼,检测品种均侧重于2019年5月1日前列管的25种芬太尼和2种前体物质,存在检测品种覆盖范围窄的缺点。

整类芬太尼类物质检测的难点在于一是品种繁多,目前全球公布已发现的芬太尼品种近150种,且目前中国整类列管,二三十种芬太尼类物质的检测无法覆盖整类检测的要求;二是芬太尼类物质标准品因为其高度敏感性、高度管控性和价格极其昂贵,实验室根本无法购买到全部标准品。本方法尝试将整类近150种芬太尼按相对分子质量、分子结构和碎裂规律进行分类。将化合物相对分子质量按顺序排列,相对分子质量不同的化合物分子结构相近、二级碎片的碎裂规律可能相似的归为一类,如乙酰、丙酰、丁酰芬太尼等归为一类,选取1种购买标准品,其他不能归类的相对分子质量不同、分子结构差异较大化合物都单独购买标准品。相对分子质量相同的异构体类化合物,分子结构和碎裂规律相近的,归为一类购买1种标准品,但分子结构和碎裂规律可能差异较大的每一类也需选取1种购买标准品,按此归类思路将近150种芬太尼分成29大类,购买到代表性的29种芬太尼标准品。本方法除对29种芬太尼进行准确定性和定量检测外,根据29种芬太尼类物质的二级谱图,解析每一个碎片的碎裂途径,详细总结了整类芬太尼物质的碎裂规律,尝试在无标准品的情况下,为芬太尼类物质的整类筛查检测提供参考。

## 1 实验部分

### 1.1 仪器、试剂与材料

TripleTOF 5600+液相色谱-四极杆/飞行时间质谱仪和Analyst TF1.7工作站(美国AB Sciex公司); MSA225S-1CE-DA分析天平(德国Sartorius公司,感量0.01 mg); JY3002分析天平(上海精密科学仪器公司,感量0.01 g); MS 3 Digital涡旋混合器(德国IKA公司); PRO VF-T超纯水系统(德国Sartorius公司); Hermle Z323K离心机(德国Hermle公司);移液器(10~100 μL、20~200 μL、100~1000 μL,德国Eppendorf公司)。

乙腈和甲醇(色谱纯)购自美国Thermo Scientific公司;甲酸(色谱纯)购自北京迪科马科技有限公司。

29种芬太尼标准品(见表1):硫代芬太尼、*α*-甲基硫代芬太尼、4-甲氧基乙酰芬太尼、4-甲氧基丙烯酰芬太尼、4-甲氧基甲氧乙酰芬太尼、4-甲氧基四氢呋喃芬太尼和庚酰芬太尼固体标准品,纯度均大于95%,购自上海原思标物科技有限公司;其余22种芬太尼标准品(100 mg/L,溶剂为甲醇)购自天津阿尔塔科技有限公司。

**表1:** 29种芬太尼类物质的质谱参数和保留时间

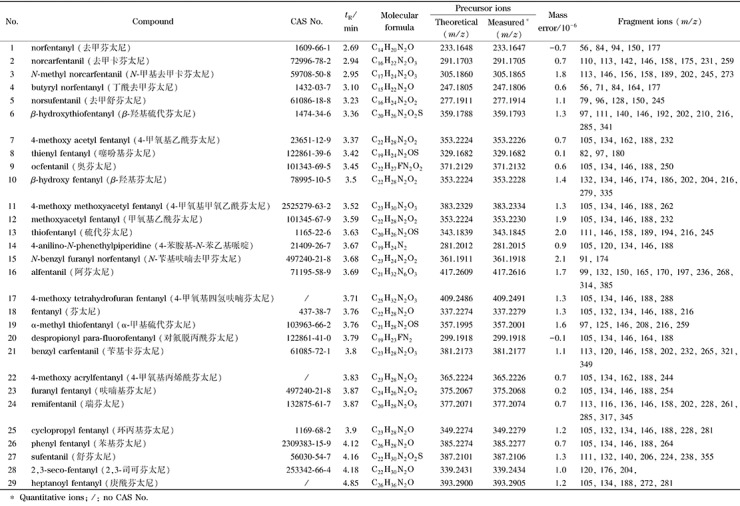

### 1.2 标准溶液配制

分别准确吸取22种100 mg/L液体标准溶液1 mL,用甲醇定容至10 mL,配成质量浓度为10 mg/L的标准储备溶液;分别准确称取7种固体标准品1 mg(精确至0.01 mg),用甲醇定容至10 mL,配成质量浓度为100 mg/L的标准储备溶液,于4 ℃避光保存。

分别吸取上述29种标准储备溶液适量,置于10 mL容量瓶中,用乙腈定容至刻度,配成质量浓度为200 μg/L的混合标准储备溶液,于4 ℃避光保存。将混合标准储备溶液用10%乙腈水溶液稀释成适当浓度的混合标准工作溶液,于4 ℃避光保存。

### 1.3 样品前处理

药品和白色粉末类、蛋白质和乳饮料类:称取试样0.2 g(精确至0.01 g,固体样品称量前需捣碎或研磨等混合均匀,液体样品称量前需摇匀),置于50 mL塑料离心管中,加入10 mL乙腈提取溶液,在涡旋器上涡旋2 min,以4 000 r/min离心5 min,取上清液2 mL过滤膜(0.20 μm,有机相),待测。

含糖固体或粉末类、饮用水类、果蔬饮料类、保健饮料类、茶饮料类、酒类:提取溶液为10 mL 10%乙腈水溶液,其余操作同上。

### 1.4 分析条件

1.4.1 色谱条件

色谱柱:Kinetex C18柱(100 mm×2.1 mm, 2.6 μm);柱温:40 ℃;流动相A为0.08%甲酸水溶液,流动相B为乙腈;流速:0.4 mL/min。梯度洗脱程序:0~5.0 min, 5%B~80%B; 5.0~8.0 min, 80%B; 8.0~8.1 min, 80%B~5%B; 8.1~10.5 min, 5%B。进样量:10 μL。

1.4.2 质谱条件

离子化模式:电喷雾电离(ESI)源,正离子模式;质谱校正方式:甲酸钠溶液外标法校正;校正周期:甲酸钠校正液和其他测试溶液穿插进样(每进一针标准溶液或样品前需要先进一针甲酸钠溶液校正质谱质量数);采集模式:一级质谱和信息依赖触发的二级质谱同时采集(TOF MS-IDA-TOF MS/MS);扫描范围:*m/z* 50~650;电喷雾电压(IS): 5500 V;离子源温度(TEM): 550 ℃;雾化气压力(GS1): 345 kPa;辅助气压力(GS2): 414 kPa;气帘气压力(CUR): 276 kPa;去簇电压(DP): 80 V;碰撞能(CE): (30±5) V。

### 1.5 定性检测

1.5.1 高分辨质谱库建立

输入29种芬太尼类物质的化学式和中英文名称,由高分辨质谱软件计算得到每个化合物母离子的理论精确质量数([M+H]^+^),构建为一级化合物谱库,见表1。

分别单独采用29种芬太尼类物质的标准工作溶液,在IDA-TOF MS/MS模式下采集其二级碎片离子谱图,将其导入高分辨质谱库,构建为二级化合物谱库,29种化合物的二级离子信息见表1。

1.5.2 鉴别

在TOF MS模式下(一级),样品溶液中待测物保留时间与对应浓度标准溶液的保留时间偏差在±2.5%之内;待测物母离子精确质量数实测值与理论值偏差小于或等于5×10^-6^,则可初步判断试样中含有该种芬太尼类物质。

1.5.3 确认

对于初步鉴别出的阳性芬太尼类物质,在IDA-TOF MS/MS模式下检测其二级碎片离子,如果至少有2个及以上丰度较高的碎片离子与谱库中相对应碎片离子的相对丰度一致,即偏差不超过表2中规定的范围,且待测物子离子二级碎片离子与二级谱库的匹配度得分(library score)在60分以上(匹配时二者CE值应确保相同)的情况下,可判定为试样中存在该种芬太尼类物质。

**表 2:** 确认分析时相对离子丰度的最大允许偏差

Relative ion abundance/%	Maximum allowable deviation/%
>50	±20
20-50	±25
10-20	±30
≤10	±50

## 2 结果与讨论

### 2.1 芬太尼类物质的碎裂规律

根据29种化合物的二级质谱图,对每个化合物的每个碎片进行质谱解析,从而总结出芬太尼类化合物的一般碎裂规律,具体如下。其中芬太尼、硫代芬太尼和舒芬太尼的二级质谱图及其每个碎片离子的碎裂机理见图1。

**图 1:**
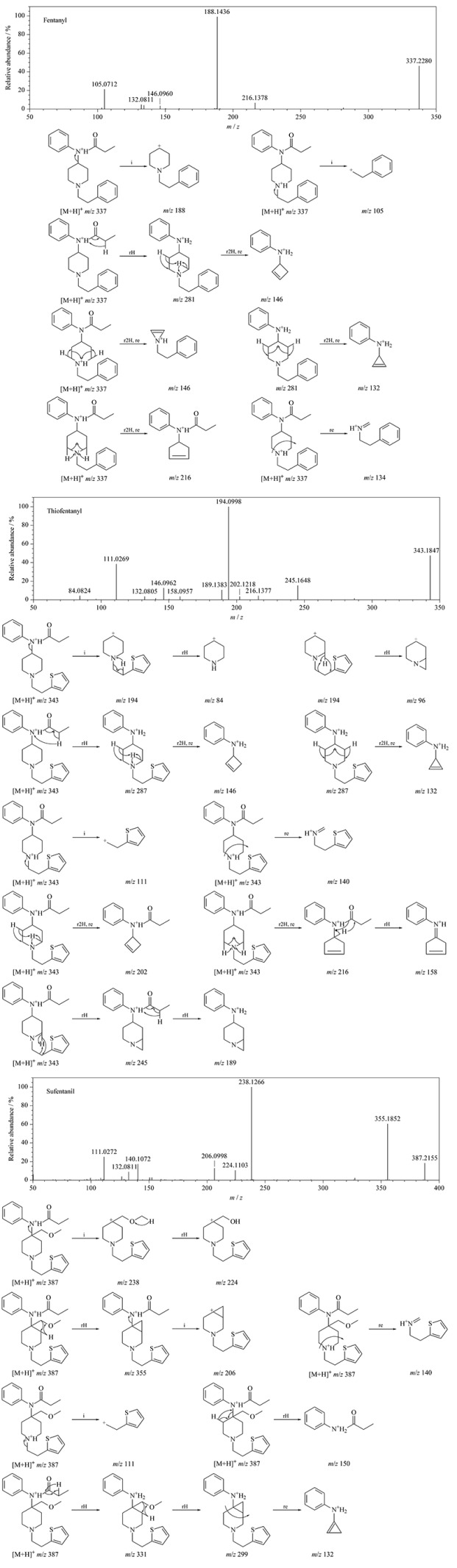
芬太尼、硫代芬太尼和舒芬太尼的二级质谱图及其碎裂机理

(1)芬太尼类化合物母离子稳定性非常好,绝大多数化合物在CE值达到30 V后,母离子才能有效碎裂。

(2)上述3种芬太尼诱导断裂(i)形成的*m/z* 188、105、194、111、238离子丰度比都较高,诱导断裂均来自酰胺基和哌啶基,且酰胺基的诱导能力明显强于哌啶基。除哌啶基对位含有其他取代基的化合物外,绝大多数芬太尼物质诱导断裂均为主要断裂方式,丰度比最高的碎片均由诱导断裂而来。

(3)芬太尼和硫代芬太尼中*m/z* 281、287的碎片离子是发生单H重排(rH)失去丙酰基的碎片离子,在庚酰芬太尼和环丙基芬太尼等化合物中均有类似的重排发生,该类离子丰度比虽不高,但具有特异性和规律性,丙酰基、庚酰基等直链烷烃取代基和环丙酰基等环烷烃类取代基均有类似特异性离子出现,直链烷烃取代基碳数越多,离子丰度越高,例如庚酰芬太尼中*m/z* 281离子丰度比明显高于芬太尼。

(4)芬太尼和硫代芬太尼中*m/z* 216、146、132碎片离子均为发生双H重排(r2H)后双键断裂形成的碎片离子,双H重排离子虽然丰度比也不高,也同样具有特异性和规律性,绝大多数芬太尼都含有该类离子,特别是双H重排得到的*m/z* 216碎片离子,对于酰胺上取代基不同的化合物通过双H重排得到的碎片则会不同,具有明显的特异性,有助于确定取代基的基团组成,与之相似碎裂规律形成的离子有庚酰芬太尼*m/z* 272、环丙基芬太尼*m/z* 228、苯基芬太尼*m/z* 264和甲氧基乙酰芬太尼*m/z* 232等的碎片离子。

(5)上述3个化合物中*m/z* 134、140均为消除反应(re)产物离子,苯乙基取代基比噻吩乙基取代基更容易发生消除反应(丰度比更高),消除反应也是该类化合物中非常普遍的碎裂方式。

(6)对比研究29种芬太尼二级碎片离子,*m/z* 146、134的碎片是苯乙基取代类芬太尼均会产生的两个碎片,具有显著的规律性。

(7)除上述3种芬太尼外,舒芬太尼这类在哌啶基对位含有甲氧基取代基的化合物碎裂离子均非常多,诱导断裂离子不一定是丰度比最高的离子,更容易发生单H重排失去CH_3_OH中性分子的碎裂模式,形成离子的*m/z*为355。类似的还有瑞芬太尼这类在哌啶基团对位含有COOCH_3_取代基的化合物,碎裂离子也较多,也更容易发生单H重排失去HCOOCH_3_或CH_3_OH中性分子的碎裂模式,形成离子的*m/z*为317或345。

(8)*β*-羟基芬太尼、*β*-羟基硫代芬太尼这类含有羟基取代基的化合物,具有显著特征的脱水离子产生,形成的离子*m/z*分别为335和341。

根据上述总结的芬太尼物质碎裂规律和特点,可以尝试辅助进行整类芬太尼类物质的筛查检测。

步骤1:通过查阅文献和资料,尽可能多搜集整类芬太尼类物质,建立收集到的芬太尼类物质一级谱库,完成非靶标筛查至靶标筛查的转换,再将未知样品LC-QTOF-MS检测后,进行一级谱库筛查,筛查是否含有精确分子量和同位素分布均满足要求的疑似阳性芬太尼类物质。

步骤2:如有疑似阳性芬太尼类物质,根据该化合物结构式和上述碎裂规律,可以大致推导出该化合物的几个主要碎片离子,再与实际得到的二级碎片离子进行比较,判断是否一致;也可以反向对比,采用实际得到的该疑似芬太尼二级碎片离子,根据上述规律研究每个碎片离子是否符合该疑似化合物的碎裂规律。通过这两个步骤可以辅助判定该疑似化合物是否为阳性。

例如:假设根据步骤1筛查出疑似物4-氯环丁基芬太尼(不常见品种),参照本文29种芬太尼中与其结构和碎裂机理类似的芬太尼、环丙基芬太尼和对氟脱丙酰芬太尼3个化合物的二级碎片和上述碎裂机理规律的总结,综合比较和推理,可以从理论上推测出4-氯环丁基芬太尼大概率含有*m/z* 188、105、146、276、134二级碎片离子,再与实际检测获得的二级碎片离子进行比对,判断是否一致。或者反向比对,通过实际得到的二级碎片离子,根据上述裂解规律总结研究每个碎片离子是否都符合裂解规律,并都能合理解释,也可判定。

### 2.2 色谱条件的优化

2.2.1 色谱柱

同分异构体因相对分子质量相同,若色谱峰重合,采用高分辨质谱无法进行准确定性和定量,需要HPLC对异构体进行分离。29种芬太尼物质中,*β*-羟基芬太尼、甲氧基乙酰芬太尼和4-甲氧基乙酰芬太尼属于同分异构体且分离比较困难。本实验选取10根不同规格和型号的色谱柱(Kinetex C18(100 mm×2.1 mm, 2.6 μm)、Atlantis dC18(150 mm×2.1 mm, 3 μm)、Poroshell EC-C18(150 mm×3.0 mm, 2.7 μm)、ZORBAX Eclipse PlusC18(150 mm×3.0 mm, 3.5 μm)、BEH C18(50 mm×2.1 mm, 1.7 μm)、ZORBAX Eclipse XDB-C18(150 mm×3.0 mm, 3.5 μm)、Atlantis T3(150 mm×2.1 mm, 3 μm)、Xselect CSH C18(150 mm×3.0 mm, 3.5 μm)、Kinetex EVO C18(100 mm×2.1 mm, 2.6 μm)和Sunfire C18(150 mm×2.1 mm, 5 μm)),尝试分离这3种化合物。除研究3种异构体分离外,同时考察了出峰最早的去甲芬太尼和出峰最晚的庚酰芬太尼在10根不同色谱柱上的峰形和保留能力。采用不同色谱柱时,在1.4.1节色谱条件下,5种芬太尼的提取离子色谱图见图2。在不同色谱柱上,5种芬太尼出峰顺序依次为去甲芬太尼、4-甲氧基乙酰芬太尼、*β*-羟基芬太尼、甲氧基乙酰芬太尼和庚酰芬太尼。

**图 2:**
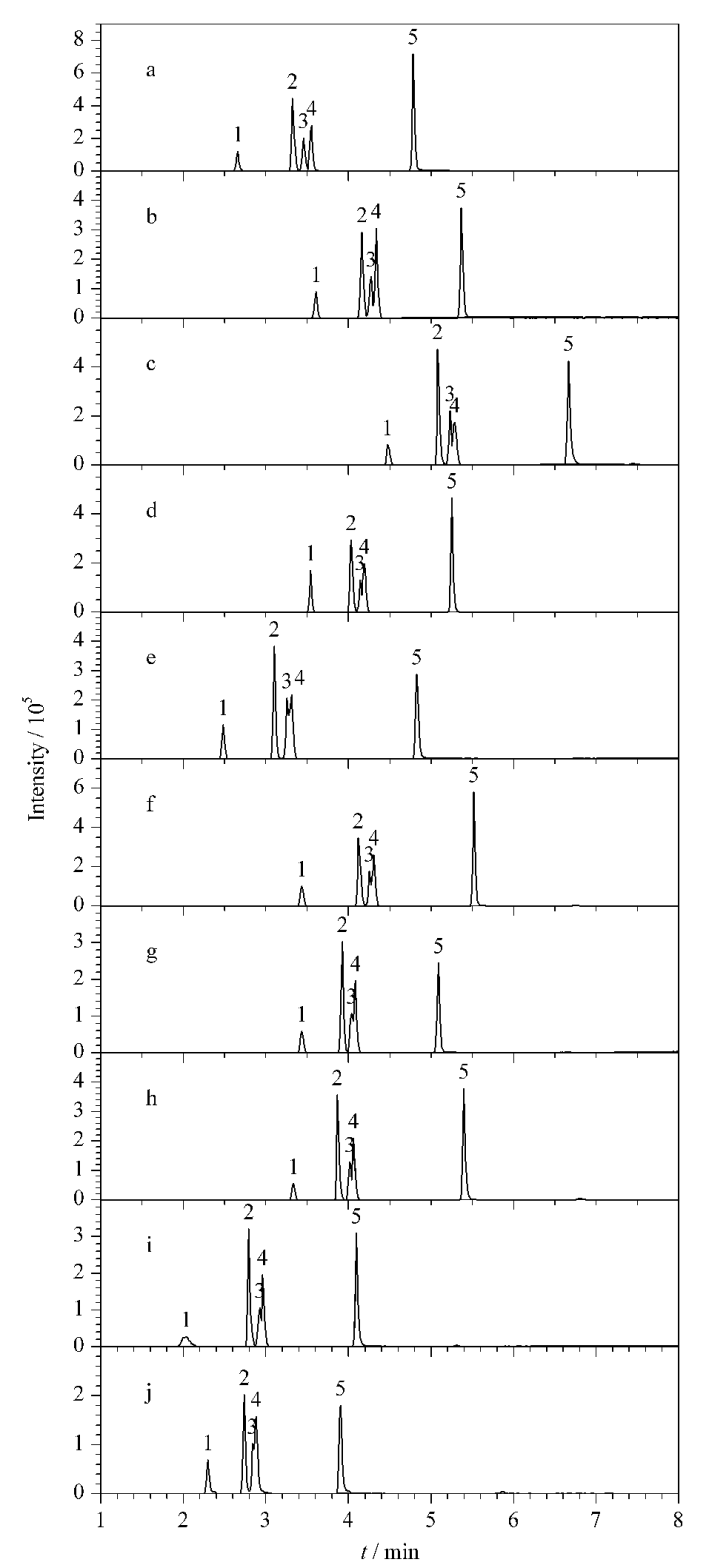
5种芬太尼在不同色谱柱上的提取离子色谱图

由图2可知,采用Kinetex C18色谱柱(100 mm×2.1 mm, 2.6 μm)时,3种异构体分离效果最好,接近基线分离,其他色谱柱分离情况均不理想。同时在该色谱柱上,去甲芬太尼和庚酰芬太尼峰形良好,二者均无明显的峰拖尾和峰展宽,保留时间也比较理想,其他24种化合物在该色谱柱上也峰形良好,所有化合物容量因子均介于1.5~5.0之间,效果较好。因此本方法最终选择Kinetex C18色谱柱(100 mm×2.1 mm, 2.6 μm)。

2.2.2 流动相起始比例

在采用Kinetex C18色谱柱(100 mm×2.1 mm, 2.6 μm)、流速为0.4 mL/min的条件下,去甲芬太尼、丁酰去甲芬太尼和去甲舒芬太尼混合标准溶液(50 μg/L)分别按5%乙腈和10%乙腈起始比例梯度洗脱程序进行测试分析(见图3)。实验表明,起始比例不同,对出峰较早、极性较强的上述3种化合物峰形影响较大。采用10%乙腈起始比例梯度洗脱程序,3种化合物半峰宽均较宽,去甲芬太尼的峰展宽更显著,5%乙腈起始比例梯度洗脱程序3种化合物,色谱峰半峰宽均较窄,峰形更佳,因此本方法选取5%乙腈起始比例的梯度洗脱程序。在优化后确定的色谱条件下,29种芬太尼的保留时间见表1,提取离子色谱图见图4。

**图 3:**
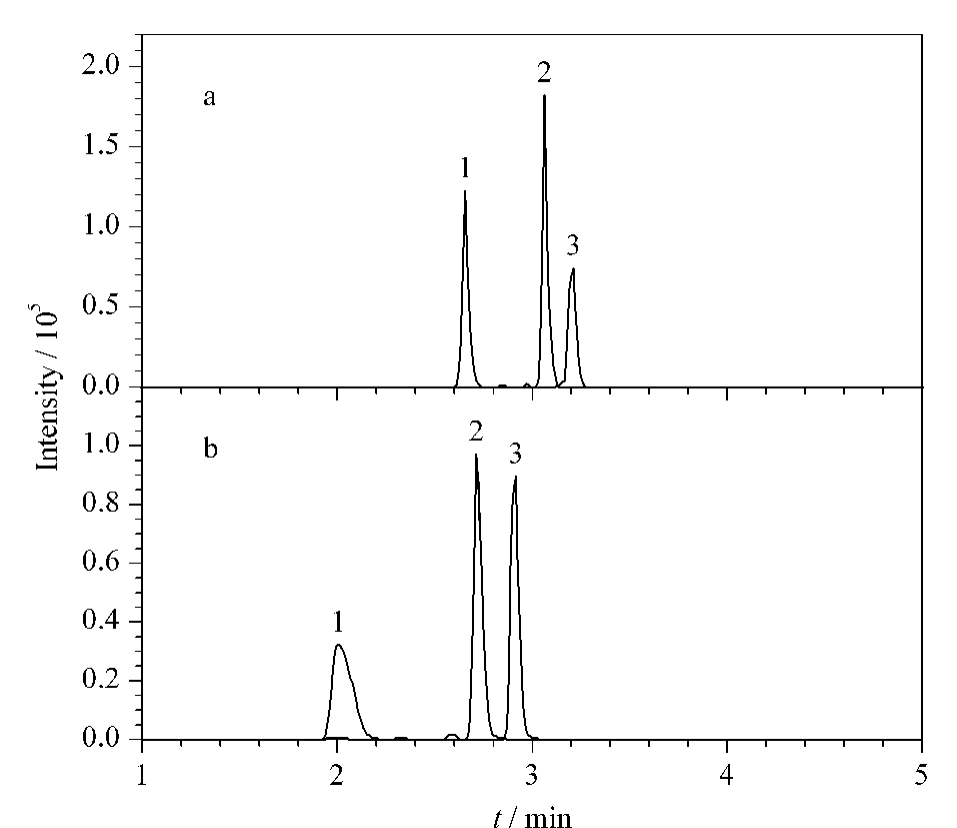
不同梯度洗脱程序下3种芬太尼的提取离子色谱图

**图 4:**
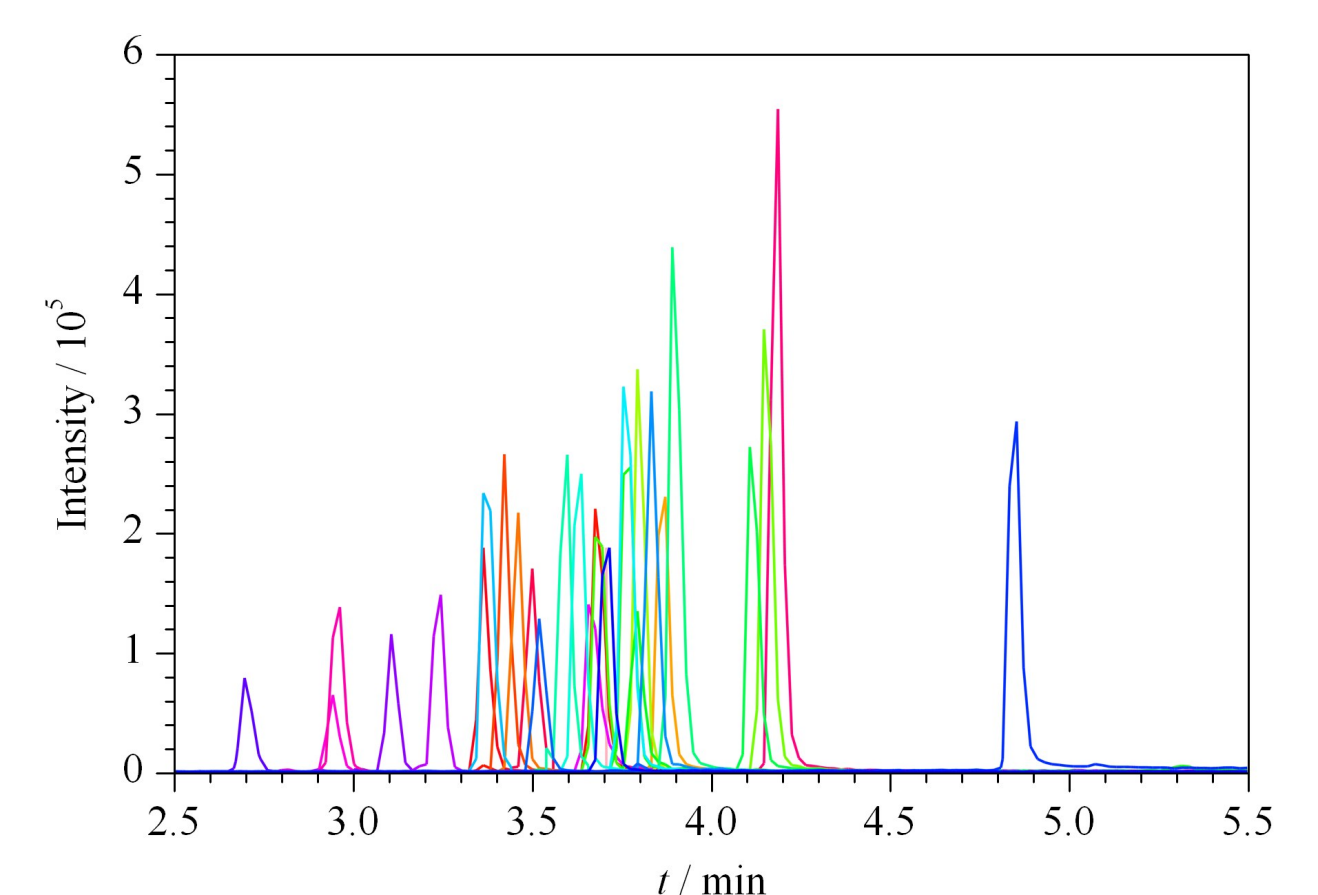
29种芬太尼混合标准溶液(10.0 μg/L)的提取离子色谱图

### 2.3 基质效应

本方法前处理采用直接提取稀释法,样品中可能会存在一定的基质效应干扰。本实验对降糖药、露露、葡萄糖粉、珍露保健饮料和巧克力5种基质进行了考察,并以目标物在空白基质液中的峰面积与溶剂中峰面积的百分比来评估基质效应。当结果为80%~120%时,表明无基质效应;高于120%,表明有基质增强效应;低于80%,则表明有基质抑制效应。结果显示,29种芬太尼在5种基质中的基质效应为75.2%~119.5%,无基质增强效应,存在一定的基质抑制效应,但基质抑制效应不明显。考虑到芬太尼类物质检测基质较特殊,空白基质不易获得,基质匹配定量在日常检测中的应用会受到限制。因此,本实验未采用基质匹配进行定量,采用溶剂标准溶液进行定量。

### 2.4 方法学考察

2.4.1 线性范围

将29种芬太尼混合标准储备溶液,用10%乙腈水溶液稀释成质量浓度为1、2、5、10和20 μg/L的标准工作溶液,在1.4节条件下进行检测,以峰面积对标准溶液中各组分的质量浓度绘制工作曲线。29种芬太尼在1~20 μg/L范围内呈线性关系,相关系数(*r*)均大于0.995。

2.4.2 检出限、回收率、精密度

本方法按照信噪比(*S/N*)≥3确定检出限(LOD),均为0.01 mg/kg。根据欧盟文件SANTE/11813/2017规定,定量限(LOQ)为加标回收试验中回收率和RSD均满足性能要求(回收率70%~120%和RSD≤20%)的最低加标水平。

实验选取空白降糖药、露露、葡萄糖粉、珍露保健饮料和巧克力样品进行0.05、0.1和0.5 mg/kg 3个水平的加标回收试验,每个水平做6个平行样品(*n*=6),按本方法进行检测,加标回收率和精密度数据见表3。结果表明,29种芬太尼在5种基质中回收率为85.2%~112.9%,RSD为1.9%~19.8%,说明方法准确度高,重复性好,适用于药品类、含糖固体或粉末类、饮料类、饮用水类和酒类等样品的检测。

**表 3:** 29种芬太尼的加标回收率和相对标准偏差(*n*=6)

No.	Spiked level/ (mg/kg)	Hypoglycemic drugs			Lulu drinks			Glucose powder			Zhenlu health drink			Chocolate		
		Recovery/ %	RSD/ %		Recovery/ %	RSD/ %		Recovery/ %	RSD/ %		Recovery/ %	RSD/ %		Recovery/ %	RSD/ %	
1	0.05	100.1	5.9		102.8	5.9		111.0	6.2		112.8	5.0		111.0		9.3
	0.1	92.6	5.4		90.2	2.6		90.4	4.9		90.9	4.7		90.2		6.0
	0.5	97.3	3.0		94.4	3.6		96.8	2.7		93.5	3.5		85.2		3.5
2	0.05	92.3	9.6		108.1	17.6		92.8	9.0		94.2	12.3		94.3		12.5
	0.1	93.0	19.0		101.5	10.5		92.9	18.9		94.2	18.6		96.7		10.2
	0.5	94.1	3.3		95.5	5.3		94.4	4.0		93.1	2.3		91.3		8.7
3	0.05	96.6	8.9		108.2	5.9		96.5	9.7		96.4	8.6		109.1		15.5
	0.1	100.5	5.4		94.8	12.0		101.4	5.6		101.1	10.3		106.0		12.3
	0.5	96.1	2.8		96.8	4.2		96.1	2.7		96.2	2.2		99.3		5.8
4	0.05	97.1	12.8		106.2	5.0		90.1	12.5		97.8	13.1		95.5		11.3
	0.1	100.6	8.3		105.8	4.3		99.9	8.7		102.0	8.6		97.6		5.6
	0.5	98.3	5.5		97.1	3.2		99.1	5.5		98.2	3.0		98.0		6.6
5	0.05	94.9	6.3		93.0	4.1		95.5	6.1		94.8	7.2		100.2		9.9
	0.1	102.6	2.6		105.0	4.9		102.7	3.0		104.0	5.4		101.1		7.7
	0.5	97.1	5.6		102.3	5.0		97.4	5.5		100.5	5.1		95.0		4.4
6	0.05	103.1	3.5		94.9	6.1		101.0	4.2		101.5	3.8		103.0		6.6
	0.1	102.6	3.8		92.8	5.4		102.9	4.0		101.8	3.8		103.1		4.3
	0.5	101.9	4.2		107.3	6.9		102.5	3.9		102.8	3.4		100.2		3.7
7	0.05	100.7	2.4		109.4	2.8		106.3	2.9		106.1	6.4		103.2		4.5
	0.1	98.2	2.1		95.9	3.7		98.4	2.3		99.4	3.4		96.0		3.8
	0.5	107.7	6.0		97.6	7.2		90.5	5.7		89.7	4.0		90.8		3.7
8	0.05	92.9	8.7		99.3	7.4		94.2	7.3		95.4	10.7		96.5		7.7
	0.1	99.6	9.0		101.2	5.7		101.2	8.9		105.0	8.0		98.2		8.1
	0.5	102.4	6.3		97.6	3.1		103.1	4.4		102.5	4.3		103.1		5.0
9	0.05	91.2	19.8		97.1	13.7		93.6	18.2		90.8	17.7		97.7		16.0
	0.1	96.0	4.7		101.4	6.3		96.4	4.9		99.4	3.1		92.1		5.9
10	0.05	93.7	3.4		89.6	6.0		93.5	3.7		92.6	3.8		100.9		8.7
	0.1	92.0	3.3		93.4	3.9		92.7	3.1		91.2	4.0		101.2		8.3
	0.5	98.9	6.3		98.4	4.7		99.1	5.4		96.0	5.0		96.5		4.0
11	0.05	91.2	12.4		96.7	6.3		89.9	12.9		91.1	13.8		91.7		5.5
	0.1	101.9	3.0		91.7	7.1		103.6	3.5		104.2	2.5		98.0		4.1
	0.5	102.6	4.0		95.8	5.7		102.2	3.7		102.2	3.9		99.1		6.0
12	0.05	100.8	4.1		88.8	2.6		112.4	4.3		111.7	5.5		91.4		5.4
	0.1	96.7	6.0		100.9	4.8		97.0	8.2		97.6	3.4		95.1		4.1
	0.5	104.2	5.0		108.7	5.8		104.2	5.1		105.8	5.0		107.2		4.4
13	0.05	90.7	11.1		102.5	8.7		89.6	10.9		92.6	15.5		91.3		15.3
	0.1	97.5	4.3		94.0	5.8		97.7	3.9		98.4	2.3		101.1		4.3
	0.5	100.5	3.4		93.8	5.0		101.5	3.9		99.3	2.8		96.7		3.0
No.	Spiked level/ (mg/kg)	Hypoglycemic drugs			Lulu drinks			Glucose powder			Zhenlu health drink			Chocolate		
		Recovery/ %	RSD/ %		Recovery/ %	RSD/ %		Recovery/ %	RSD/ %		Recovery/ %	RSD/ %		Recovery/ %	RSD/ %	
14	0.05	91.3	10.0		100.9	9.3		90.6	8.9		90.7	9.0		103.8		14.9
	0.1	102.1	4.1		100.5	5.4		102.4	3.9		102.8	5.2		99.5		6.0
	0.5	99.7	7.4		101.9	7.1		99.8	7.8		104.2	6.5		95.5		6.1
15	0.05	102.6	6.1		105.2	7.1		98.4	6.6		100.9	8.1		97.7		9.2
	0.1	109.1	5.0		99.0	4.2		107.6	6.1		104.1	4.4		98.0		8.0
	0.5	102.3	6.2		94.4	2.3		102.7	4.5		97.6	1.9		95.1		3.9
16	0.05	98.2	8.5		108.1	5.5		91.9	7.3		102.6	11.5		92.3		8.9
	0.1	95.3	2.6		96.8	2.7		95.7	3.9		94.9	3.4		95.0		4.2
	0.5	99.4	7.7		101.9	2.3		104.9	2.7		96.9	5.0		96.1		3.1
17	0.05	96.7	15.1		90.9	8.3		95.1	14.9		100.4	16.2		93.3		9.1
	0.1	98.1	9.5		94.9	9.7		96.9	9.2		105.3	9.8		102.1		5.7
	0.5	104.3	5.9		101.7	6.8		102.8	4.7		98.2	6.0		98.4		6.6
18	0.05	97.7	11.4		100.4	5.9		97.3	11.0		99.2	9.5		100.3		6.9
	0.1	100.8	5.0		94.7	2.9		101.4	6.0		101.0	3.9		101.1		4.3
	0.5	103.4	6.1		88.5	5.4		102.9	6.5		97.6	3.4		94.0		5.7
19	0.05	105.6	9.0		92.8	7.8		107.5	8.5		105.6	11.3		108.2		9.8
	0.1	102.6	6.7		94.3	4.1		103.2	6.1		98.8	3.5		95.5		5.5
	0.5	90.7	4.0		98.6	6.3		91.5	2.8		91.1	4.4		90.2		3.3
20	0.05	96.4	19.1		102.6	15.0		96.2	18.1		93.8	18.8		94.7		17.2
	0.1	104.9	5.0		96.9	3.2		106.4	5.1		103.2	3.2		105.2		6.7
	0.5	101.5	8.9		99.2	8.6		105.4	5.0		106.1	8.5		107.2		7.8
21	0.05	96.4	11.3		99.5	5.8		94.6	10.5		94.7	8.8		92.3		10.0
	0.1	99.4	9.3		101.0	4.5		98.9	8.7		99.6	8.0		99.2		6.8
	0.5	99.7	6.9		93.4	7.4		99.7	7.2		100.2	5.8		94.2		6.1
22	0.05	96.3	8.4		105.0	5.8		95.0	8.2		97.5	13.0		94.4		10.2
	0.1	101.4	6.1		98.0	6.8		102.0	6.1		102.3	5.8		97.7		5.9
	0.5	90.5	4.1		98.0	4.0		90.1	3.9		95.9	3.7		93.2		3.8
	0.5	101.2	3.8		96.3	4.2		102.5	2.5		97.2	3.7		93.9		4.9
23	0.05	97.5	4.3		96.5	12.6		95.2	4.8		98.2	4.8		96.2		10.2
	0.1	101.6	8.6		98.8	5.5		101.8	8.5		96.4	6.0		102.3		8.1
	0.5	91.2	5.1		104.1	4.0		91.8	5.3		89.5	5.2		89.9		4.7
24	0.05	97.3	12.8		100.3	15.4		100.2	15.5		102.7	15.2		102.0		14.4
	0.1	97.2	15.6		103.3	10.1		99.8	13.5		97.3	12.0		102.3		13.1
	0.5	90.6	4.7		104.6	8.2		93.7	4.7		93.1	3.4		93.9		5.6
25	0.05	86.1	3.9		102.5	12.5		86.8	4.2		89.3	8.0		90.5		7.4
	0.1	97.8	2.2		102.8	7.7		98.0	2.5		90.6	2.1		90.6		2.8
	0.5	93.7	4.8		97.2	4.6		94.4	4.3		91.0	4.7		92.5		4.9
26	0.05	94.2	17.2		103.6	15.9		94.3	17.3		103.2	16.2		95.9		11.3
	0.1	101.2	5.2		101.0	3.9		100.8	4.8		96.4	3.1		97.7		4.0
	0.5	103.8	7.1		90.5	6.2		98.4	4.7		94.1	5.7		92.6		4.2
27	0.05	96.8	6.4		97.9	7.4		98.5	7.1		107.4	10.0		108.2		11.7
	0.1	102.7	9.8		97.1	3.8		103.3	9.8		101.4	7.1		102.9		8.8
	0.5	99.7	3.1		100.0	5.3		100.3	2.9		96.0	2.8		95.5		10.3
28	0.05	102.7	4.2		99.2	9.1		102.6	4.2		101.3	8.7		102.5		9.8
	0.1	88.6	3.1		95.6	5.2		88.6	3.2		95.3	3.2		103.2		8.7
	0.5	94.7	6.5		95.3	3.5		97.2	5.6		89.1	4.3		98.2		3.3
29	0.05	104.1	11.6		101.3	18.5		104.1	11.2		112.9	13.0		108.2		14.4
	0.1	102.2	14.2		93.0	13.2		103.4	14.5		93.8	8.9		94.0		9.1
	0.5	95.2	7.7		103.6	4.9		95.9	7.8		101.3	5.1		96.2		4.9

Nos. 1-29 were the same as that in Table 1.

由上述可知,本方法最低加标水平为0.05 mg/kg, 29种芬太尼在5种基质中平均回收率和RSD均满足要求,且信噪比(*S/N*)>10,因此确定本方法LOQ均为0.05 mg/kg。

### 2.5 实际样品的检测

建立的方法应用于大窑湾海关和大连邮局海关可疑样品的检测,共检测葡萄糖粉(白色粉末)样品11批次、纯净水样品2批次、旧报纸样品(报纸样品可参照药品和白色粉末类、蛋白质和乳饮料类步骤进行前处理)2批次、可疑白色药片3批次和珍露保健饮料(黑色液体)1批次,检测结果均为阴性。

## 3 结论

本文建立了飞行时间高分辨质谱定性和定量检测29种芬太尼类物质的分析方法,该方法检测速度快,定性准确,灵敏度高,实用性强,能够满足公安缉毒、刑侦、邮局海关和机场海关等对芬太尼监控的检测技术要求,具有较好的应用价值。本文详细研究了29种芬太尼化合物每个碎片离子的碎裂机理,总结出芬太尼类化合物的碎裂规律和特点,对整类芬太尼类物质的筛查检测,具有一定的参考价值。
